# High prevalence and significant ethnic differences in actionable HbA_1C_ after gestational diabetes mellitus in women living in Norway

**DOI:** 10.1186/s12916-022-02515-w

**Published:** 2022-09-23

**Authors:** Archana Sharma, Ingrid Nermoen, Elisabeth Qvigstad, Anh T. Tran, Christine Sommer, Naveed Sattar, Jason M. R. Gill, Hanne L. Gulseth, Stina T. Sollid, Kåre I. Birkeland

**Affiliations:** 1 Department of Endocrinology, Akershus University Hospital, University of Oslo, 1478 Lørenskog, Norway; 2 Institute of Clinical Medicine, University of Oslo, Oslo, Norway; 3 Department of Endocrinology, Morbid Obesity and Preventive Medicine, Oslo University Hospital, Oslo, Norway; 4 Institute of Health and Society, Department of General Practice, University of Oslo, Oslo, Norway; 5 Institute of Cardiovascular and Medical Sciences, University of Glasgow, BHF Glasgow Cardiovascular Research Centre, 126 University Place, Glasgow, G12 8TA UK; 6 Norwegian Institute of Public Health, Oslo, Norway; 7 Department of Medicine, Drammen Hospital, Vestre Viken Health Trust, Drammen, Norway

**Keywords:** Gestational diabetes mellitus, Glucose metabolism disorders, Ethnic groups, Prevention, Overweight, Glycated haemoglobin

## Abstract

**Background:**

The type 2 diabetes risk after gestational diabetes mellitus (GDM) is twice as high in South Asian compared to European women. Current guidelines differ regarding which test to use as a screening-tool post-GDM. We aimed to identify ethnic differences in the prevalence rates and early predictors for actionable HbA_1c_ (defined as prediabetes and diabetes) short time after GDM.

**Methods:**

This cross-sectional study, enrolling South Asian and Nordic women 1–3 years after a diagnosis of GDM, was undertaken at three hospitals in Norway. We performed a clinical and laboratory evaluation including an oral glucose tolerance test (OGTT). Medical records were used to retrieve data during pregnancy. Prediabetes was classified with HbA_1c_ alone or combined with OGTT glucose measurements according to the WHO, WHO-IEC, and ADA criteria (fasting plasma glucose (FPG) 6.1–6.9 mmol/L, FPG 6.1–6.9 mmol/L and/or HbA_1c_ 42-47 mmol/mol (6.0-6.4%), and FPG 5.6–6.9 mmol/L and/or HbA_1c_ 39-47 mmol/mol (5.7-6.4%)). Ethnic differences in prevalence and predictors of glucose deterioration were assed by *χ*^2^ (Pearson) tests and logistic regression models.

**Results:**

We included 163 South Asian and 108 Nordic women. Actionable HbA_1c_ levels were highly prevalent and more so among South Asian than Nordic women (WHO-IEC-HbA_1c_: 25.8% vs. 6.5% (*p* ≤ 0.001), ADA-HbA_1c_: 58.3% vs. 22.2% (*p* ≤ 0.001)). Although adding OGTT-data gave higher combined prevalence rates of prediabetes and diabetes (WHO: 65.6% vs. 47.2% (*p* ≤ 0.05), WHO-IEC: 70.6% vs. 47.2% (*p* ≤ 0.001), ADA: 87.8% vs. 65.7% (*p* ≤ 0.001)), the excess risk in the South Asian women was best captured by the HbA_1c_. Important predictors for glucose deterioration after GDM were: South Asian ethnicity, GDM before the index pregnancy, use of glucose-lowering drugs in pregnancy, higher age, and higher in-pregnancy fasting glucose levels.

**Conclusions:**

In women with GDM 1–3 year previously, we found high prevalence and significant ethnic differences in actionable ADA-HbA_1c_ levels, with South Asian ethnicity, GDM before the index pregnancy, and the use of glucose-lowering drugs in pregnancy as the most important risk factors. This study reinforces the importance of annual screening—preferably with HbA_1c_ measurements—to facilitate early intervention after GDM.

**Supplementary Information:**

The online version contains supplementary material available at 10.1186/s12916-022-02515-w.

## Background

Gestational diabetes mellitus (GDM) is associated with an increased risk of diabetes later in life [1, 2], particularly among South Asian women living in high-income countries [3]. The prevalence of both GDM [4, 5] and subsequent type 2 diabetes [2, 3] are twice as high in South Asian compared to European women; additionally type 2 diabetes seems to develop earlier and at a lower body mass index (BMI) in South Asian women post-GDM [6].

Accordingly, accurate diagnostic tools are important not only when considering preventive measures to counteract the development of diabetes but also to detect women at high risk of GDM in subsequent pregnancies. Several national guidelines recommend lifelong screening by glycated haemoglobin (HbA_1c_), fasting plasma glucose (FPG), or oral glucose tolerance test (OGTT) post-GDM to identify women at high-risk [7–9]. In clinical practice, however, HbA_1c_ rather than FPG or performing an OGTT are preferred, due to advantages as (i) less pre-analytical and day-to-day variation, and, of greater importance, (ii) the stronger association including in seminal meta-analyses between glycated haemoglobin and diabetes complications, both for retinopathy [10] and cardiovascular disease [11, 12] as compared to 2-h glucose. Further, more convenient blood sampling without the need for fasting and the (unpleasant) time-consuming OGTT is a major benefit. Notwithstanding this, available literature refers to OGTT as gold-standard test when comparing different modes for diagnosis of prediabetes and diabetes [13, 14] and suffers from variation in the use of diagnostic criteria to define GDM and prediabetes post-GDM [15]. Similarly, risk factors for prediabetes and diabetes among different ethnicities cared for in the same healthcare setting post-GDM have not been clearly characterized.

With this background, in women with previous GDM living in Norway, we investigated the impact of South-Asian and Nordic ethnicity on (1) the prevalence of prediabetes and diabetes using HbA_1c_ without and with OGTT measurements by the WHO, WHO-IEC, or ADA criteria [16–18] and (2) pre- and in-pregnancy predictors for actionable HbA_1c_ (defined as prediabetes or diabetes) 1–3 years after delivery.

## Methods

The ongoing DIAbetes in South Asians 1 (DIASA 1) study was approved by the Regional Committee for Medical and Health Research Ethics of South-Eastern Norway (reference number: 2018/689). All participants signed study-specific consent forms.

### Design, study population, and data collection

Between September 1, 2018, and November 1, 2021, the DIASA 1 cross-sectional study recruited women with a diagnosis of GDM in their last pregnancy, who delivered 12–36 (± 3) months previously at one of three hospitals in the Oslo area, Norway. The inclusion criteria were age ≥ 18 years, ethnic origin from a South Asian (Pakistan, India, Bangladesh, or Sri Lanka) or Nordic (Norway, Sweden, Denmark, Finland, or Iceland) country. Exclusion criteria were new pregnancies after the index pregnancy, exclusive breastfeeding at the time of examination, known diabetes before the index pregnancy or at the time of examination, ongoing inflammatory or serious disease, or a history of major surgical procedure < 3 months prior to inclusion. The eligible women, identified by searching medical records from the three hospitals, were recruited through an invitation letter. Additionally (as recommended by Regional Ethics Committee), the South Asian women received a telephone invitation in their native language.

At the study visit, we measured height, weight, waist, and hip circumferences [19]. Thereafter, all women underwent a two-hour 75 g OGTT between 08.00 and 10.00 am after at least 8 h fasting at their local hospitals.

Blood for glucose analysis were collected in cooled sodium fluoride tubes and kept on ice until centrifugation, and plasma was analysed at Oslo University Hospital (Aker) using enzymatic photometry (Roche Diagnostics, Mannheim, Germany). Whole-blood HbA_1c_ was analysed by high-performance liquid chromatography (Tosoh G8 analyser, Tokyo Japan). The coefficients of variation were 2.5% for glucose and 1.5–2.5% for HbA_1c_.

Clinical and biochemical data from the women obtained during the pregnancy, including the results of the 75 g OGTT performed at gestational weeks 24–28, were retrieved from medical records.

### Definitions of GDM, prediabetes, and diabetes

Until April 2017, the Norwegian healthcare system used targeted screening for GDM in high-risk groups and applied the WHO 1999 criteria (FPG ≥ 7.0 or 2-h PG ≥ 7.8 mmol/l) [16]. From April 2017, this routine changed to universal screening and modified International Association of Diabetes and Pregnancy Study Group (IADPSG) criteria with FPG 5.3–6.9 or 2-h glucose 9.0–11.0 mmol/l [7] (no 1-h glucose). For the few cases registered as GDM in pregnancy, where OGTT data were not available, we recorded FPG ≥ 5.3 mmol/L or initiating glucose-lowering drugs in pregnancy as diagnostic of GDM (*n* = 6).

The prevalence of prediabetes was assessed according to the following criteria:

1. The WHO criteria: FPG 6.1–6.9 mmol/L and/or 2-h plasma glucose 7.8–11.0 mmol/L [16]
2. The WHO-IEC criteria: FPG 6.1–6.9 mmol/L and/or 2-h plasma glucose 7.8–11.0 mmol/L and/or HbA_1c_ 42-47 mmol/mol (6.0–6.4%) [16, 17]
3. The ADA criteria: FPG 5.6–6.9 mmol/L and/or 2-h plasma glucose 7.8–11.0 mmol/L and/or HbA_1c_ 39-47 mmol/mol (5.7–6.4%) [18]

The prevalence of diabetes was assessed according to the internationally-agreed criteria: FPG ≥ 7.0 mmol/L and/or 2-h plasma glucose ≥ 11.1 mmol/L and/or HbA_1c_ ≥ 48 mmol/mol (≥ 6.5%) [18, 20]. For a clinical diagnosis of diabetes in asymptomatic individuals, two separate tests are required. In the present study, however, we accepted only one test.

### Statistical analyses

Based on a published study [21], we expected the proportion of women with prediabetes or diabetes to be 35% in South Asian and 20% in Nordic women. Accordingly, with *α* = 0.05, the study needed a total number of 324 subjects to have a statistical power of 80% to detect differences in the prevalence between the ethnic groups. However, due to higher prevalence rates, greater difference between the groups, and delayed recruitment during the COVID-19 pandemic, we performed an interim data analysis that ensured sufficient power (97%) to detect differences between the groups with the included 271 women in 2021.

Differences between groups were assessed with unpaired t-tests for normally distributed data, and with Mann–Whitney test for non-normally distributed data. *χ*^2^ (Pearson) and Fisher’s exact tests were applied to compare group proportion differences for prediabetes and diabetes. Characteristics were presented as mean (SD), or median (interquartile range, IQR), or number [%]. We calculated confidence intervals (CI) for proportions, and used plots to show the distribution of HbA_1c_ by ethnicity, and FPG by HbA_1c_.

Evaluated against the different diagnostics criteria, we compared the diagnostic performance of the FPG, HbA_1c_, and the combination of both for sensitivity, specificity, and positive and negative predictive value in the South Asian and Nordic group.

Logistic regression analyses were performed to identify determinants for actionable ADA-HbA_1c_. Age, ethnicity, glucose-lowering drugs, in-pregnancy FPG and 2-h OGTT values, pre-pregnancy BMI, gestational weight gain (GWG, calculated as the difference between weight within 4 weeks before delivery and pre-pregnancy weight), parity, education (as a proxy for socio-economic status), first-degree relatives with diabetes, and GDM before index pregnancy were entered as adjustment covariates. Covariates with *p*-values ≤ 0.25 in the univariate logistic regression analyses were included in a multivariate logistic regression analysis to find the most significant predictors for actionable HbA_1c_. The results were expressed as odds ratio (OR) with 95% CI. For all analysis, *p*-values < 0.05 were considered statistically significant, except for interactions, where a *p*-value < 0.001 was applied to reduce the number of sporadic findings as we looked for interactions among all the covariates. We used SPSS 27 and STATA 17.

## Results

Of the 1169 (398 South Asian and 771 Nordic) eligible women with a GDM diagnosis, 271 (98 Pakistani, 30 Indian, 5 Bangladeshi, 30 Sri Lankan, 101 Norwegian, 3 Swedish, 3 Danish, and 1 Icelandic) women participated. Among the South Asians, 14 were excluded due to new-onset diabetes after index pregnancy and 22 due to a new pregnancy, whilst 199 declined or were not contactable. Among the Nordic women, who were only invited by letter, reasons for non-participating were not defined (Additional file 1: Fig. S1).

### Baseline characteristics

At a median (IQR) of 16.8 (12.2) months after delivery, the South Asian group had higher parity, more first-degree relatives with diabetes, and fewer years of education than the Nordic group. BMI did not differ between the groups, but South Asian women had higher waist-to-hip ratio (WHR) at the study visit, and were younger than the Nordic women (Table 1). In total, 58.3% (*n* = 158) had prediabetes or diabetes according to the WHO criteria, and 74.2% (*n* = 201) according to the ADA criteria, whilst the ADA-HbA_1c_ criteria captured 43.9% (*n* = 119) of these women.

**Table 1:** The participants’ characteristics and demographics by ethnicity

	South Asian ***n*** = 163 [60%]	Nordic ***n*** = 108 [40%]	***P***-value
**Characteristics at study visit:**			
Age (years)	34.5 (4.1)	36.5 (4.9)	< 0.001
Time since index pregnancy (months)^a^	16.1 (12.5)	18.3 (11.2)	0.104
Weight (kg)	73.9 (14.5)	81.7 (19.6)	0.001
Height (cm)	159.5 (6.3)	166.9 (6.1)	< 0.001
BMI (kg/m^2^)	29.0 (5.3)	29.3 (6.8)	0.688
Waist circumference (cm)	97.0 (11.6)	96.1 (14.0)	0.605
Waist/hip ratio	0.91 (0.07)	0.88 (0.09)	0.033
FPG at OGTT	5.8 (0.7)	5.7 (0.8)	0.136
2-h OGTT glucose	8.7 (2.7)	7.9 (2.6)	0.013
HbA_1c_ (mmol/mol)	(39 (5))	37 (4)	< 0.001
HbA_1c_ [%]	[5.7 (2.6)]	[5.5 (2.5)]	< 0.001
Parity	2.1 (1.0)	1.7 (0.7)	< 0.001
GDM prior to the index pregnancy	51 [32]	24 [22]	0.096
1.degree relatives w/diabetes	115/155 [74]	22/91 [24]	< 0.001
Tertiary educated (college/university)	87 [53]	81 [75]	< 0.001
Years of education	14.8 (3.3)	16.8 (3.0)	< 0.001
Employed	89 [55]	99 [92]	< 0.001
**Characteristics related to index pregnancy:**			
Pre-pregnancy age (years)	33.4 (4.1)	35.2 (4.7)	< 0.001
Self-reported pre-pregnancy weight (kg)	70.6 (13.8)	79.3 (18.0)	< 0.001
Pre-pregnancy BMI (based on self-reported weight)	27.6 (5.0)	28.4 (6.2)	0.242
Ethnicity-specific pre-pregnancy overweight ± obesity (based on self-reported weight)	137 [84.0]	73 [67.6]	< 0.001
Last weight reported before delivery (kg)	80.8 (14.0)	90.0 (16.8)	< 0.001
Gestational weight gain (kg)	10.2 (5.8)	10.7 (7.1)	0.594
Pregnancy FPG at OGTT	5.5 (0.7)	5.3 (0.7)	0.017
Pregnancy 2-h OGTT glucose	9.3 (1.8)	9.0 (1.7)	0.108
Breastfeeding (months)^a^	9.4 (10)	11.0 (7.8)	0.738
Insulin ± metformin use in pregnancy	89 [55]	40 [37]	0.005
Insulin use in pregnancy	65 [40]	34 [31]	0.160

Data presented as mean (SD) or ^a^median (IQR) or number (*n*) [%]

Ethnicity-specific pre-pregnancy BMI by the WHO definition: “overweight” in the general populations as > 25 kg/m^2^ and in the Asian population as > 23 kg/m^2^ [22]

*FPG* fasting plasma glucose, *GDM* gestational diabetes mellitus, *OGTT* oral glucose tolerance test

### Prevalence of prediabetes and diabetes by HbA_1c_

Independent of criteria used, prediabetes or diabetes was found in a sizeable proportion of South Asian women post-GDM, and prediabetes was significantly more prevalent among South Asian than Nordic women (Additional file 1: Fig. S2).

**Fig. 1:**
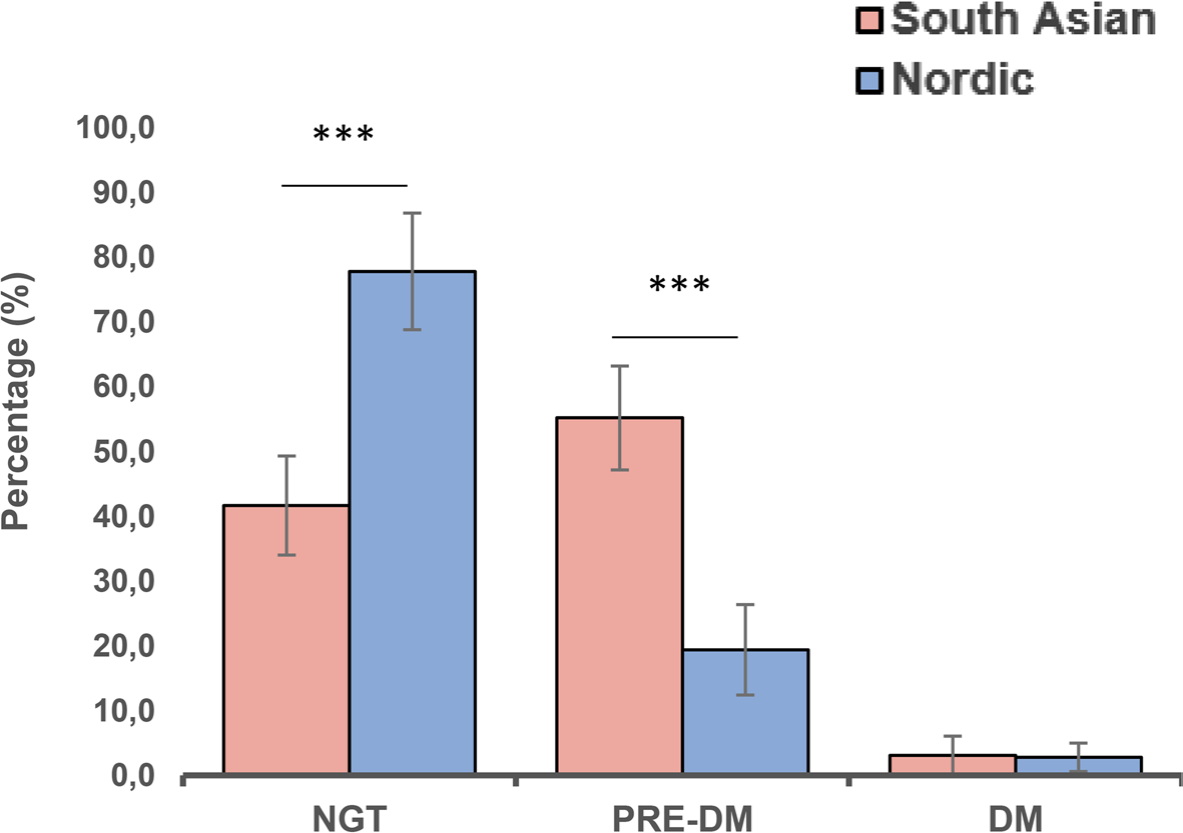
HbA_1c_-based prevalence rates (95% CI) by ethnicity. HbA_1c_-based prevalence with 95% CI of normal glucose tolerance (NGT: HbA_1c_ < 39 mmol/mol (< 5.7%)), prediabetes (PRE-DM: HbA_1c_ 39-47 mmol/mol (5.7–6.4%)) and diabetes (DM: HbA_1c_ ≥ 48 mmol/mol (≥ 6.5%)) by ethnicity using the ADA criteria. *p-value ≤ 0.05, **p-value ≤ 0.01, *** *p*-value ≤ 0.001

**Fig. 2:**
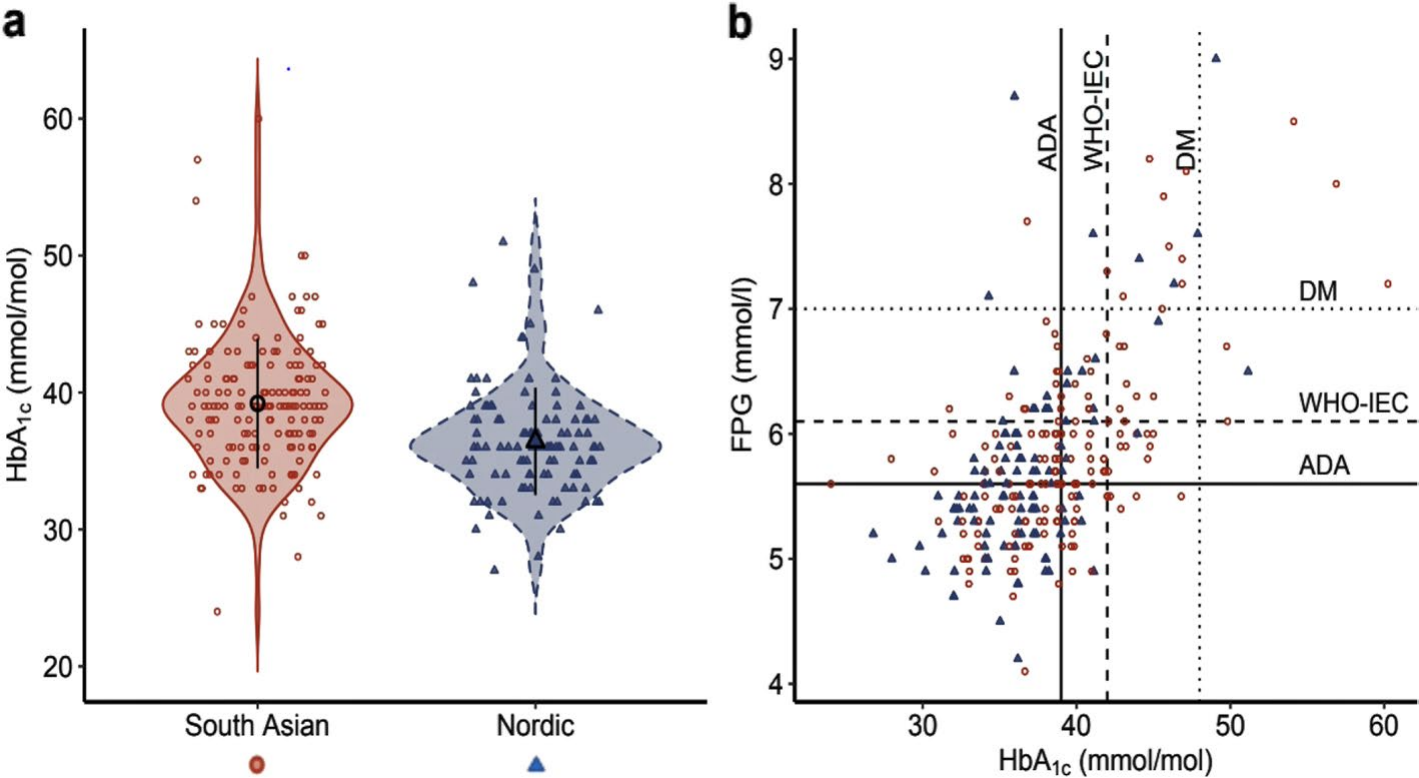
HbA_1c_ violin plot and FPG-HbA_1c_ distribution plot. **a** Violin plot showing the distribution and mean (SD) HbA_1c_ by ethnicity. **b** Distribution of fasting plasma glucose (FPG) values during oral glucose tolerance test plotted against HbA_1c._ Red circles = South Asian women, and blue triangles = Nordic women. Solid line = ADA criteria for prediabetes (FPG 5.6–6.9 mmol/L, and/or HbA_1c_ 39–47 mmol/mol (5.7–6.4%)), dotted line = WHO-IEC criteria for prediabetes (FPG 6.1–6.9 mmol/L and/or HbA_1c_ 42–47 mmol/mol (6.0-6.4%)), and dashed line = criteria for diabetes (FPG ≥ 7.0 mmol/L and/or HbA_1c_ ≥ 48 mmol/mol (≥ 6.5%))

When applying the WHO-IEC criteria for prediabetes based on HbA_1c_, 22.7% (*n* = 37) of the South Asian and 3.7% (*n* = 4) of the Nordic women qualified for this diagnosis (*p* < 0.001). With the ADA criteria, the proportions were 55.2% (*n* = 90) in the South Asian and 19.4% (*n* = 21) in the Nordic group (*p* < 0.001), respectively (Fig. 1). The distribution of individual HbA_1c_ levels, and the mean glycated haemoglobin showed higher values among South Asian women (Fig. 2a).

Further, as determined by a single HbA_1c_ measurement, the prevalence of diabetes was 3.1% (*n* =5) in South Asian and 2.8% (*n* = 3) in the Nordic group, with no difference between the groups (*p* = 0.598) (Fig. 1).

### Prevalence of prediabetes and diabetes by different diagnostic criteria

By including the OGTT data and applying the WHO criteria, 65.6% (*n* = 107) of the South Asian women could be defined as having prediabetes or diabetes. If we used the WHO-IEC criteria, the proportion increased to 70.6% (*n* = 115), whilst by the current ADA criteria, 87.8% (*n* = 143) of South Asian women had prediabetes or diabetes. The percentage of women with diabetes was 19.0% (*n* = 31). The comparable proportion of prediabetes or diabetes among the Nordic women by applying the WHO, WHO-IEC and the ADA criteria were 47.2% (*n* = 51), 47.2% (*n* = 51), and 65.7% (*n* = 71). The percentage of women with diabetes was 13.9% (*n* = 15) (Additional file 1: Fig. S2).

### Comparing different modes for diagnosis prediabetes and diabetes with the debated assumption that OGTT is gold standard

HbA_1c_ detected fewer cases of prediabetes or diabetes than when we also included OGTT glucose measurements. We, therefore, calculated the sensitivity, specificity, and positive and negative predictive value of using HbA_1c_ alone for prediabetes and diabetes diagnosis by ethnicity and different diagnostic criteria (Additional file 2: Table S1), also including different combinations of FPG and HbA_1c_ by ethnicity and different diagnostic criteria (Additional file 2: Table S2).

Compared to the compound diagnostic criteria, the sensitivity (with 95% CI) of using only HbA_1c_ for detecting prediabetes or diabetes was low, but better in South Asian than in Nordic women (WHO-IEC: 37 (28–46)% and ADA: 66 (58–74)% in the South Asian group vs. WHO-IEC: 14 (6–26)% and ADA: 34 (23–46)% in the Nordic group).

HbA_1c_ also diagnosed fewer women with diabetes as compared with the compound diagnostic criteria, but there were no differences in its sensitivity between the ethnic groups (16 (5–34)% vs. 20 (4–48)%) (Additional file 2: Table S1). By adding FPG to HbA_1c_, more women were identified with prediabetes or diabetes independent of ethnicity and criteria used. The benefits of adding HbA_1c_ to FPG in detecting prediabetes or diabetes, however, was only significant in the South Asian group (FPG: WHO-IEC: 38 (29–48)%, ADA: 71 (63–79)% vs. FPG and HbA_1c_: WHO-IEC: 55 (45–64)%, ADA: 86 (79–91)%) (Additional file 2: Table S1). A cut-off for FPG of 5.6 mmol/l and HbA_1c_ of 38 mmol/mol (5.6%) performed best in the South Asians, and similarly, FPG of 5.6 mmol/l and HbA_1c_ of 37 mmol/mol (5.5%) performed best in the Nordic group (Additional file 2: Table S2).

### Percentage of South Asian and Nordic women with actionable HbA_1c_ levels

Adding FPG to HbA_1c_ increased its sensitivity, however, the FPG-HbA_1c_ distribution plot indicated that a single ADA-HbA_1c_ captured most of the glucose values that deserved action (i.e., ADA-HbA_1c_ detected few with FPG < 5.6 mmol/L, and most of the elevated FPG levels), yet better in South Asian than Nordic women (Fig. 2b). Similarly, the number of women that needed to be tested to identify an actionable ADA-HbA_1c_ value was only one in two (95/163) for the South Asian and one in five (24/108) for the Nordic women. The corresponding number by the WHO-HbA_1c_ cut-points, however, was one in four (42/163) and one in fifteen (7/108) for the South Asian and Nordic women.

### Predictors for prediabetes or diabetes by ethnicity

We sought to identify variables before or during the index pregnancy that predicted actionable HbA_1c_ 16.8 months after delivery. As shown in Table 2, the single strongest predictor was South Asian ethnicity. In women without GDM prior to the index pregnancy, the strongest predictor was the use of glucose-lowering drugs during pregnancy, whilst this did not impact the risk in women with multiple GDM pregnancies (*p* for interaction = 0.009).

**Table 2:** Logistic regression analysis for actionable ADA-HbA_1c_ (defined as prediabetes or diabetes) after adjusting for covariates

Risk factors	Estimated coefficients	***P***-value	OR	OR (95% CI)	
				Lower	Upper
Age (years)	0.11	0.015	1.11	1.02	1.21
Ethncity	1.95	< 0.001	7.05	2.94	16.92
Glucose-lowering drugs (yes)	1.06	0.008	2.87	1.31	6.30
FPG at OGTT in pregnancy (mmol/l)	0.67	0.011	1.96	1.16	3.29
2-h OGTT glucose in pregnancy (mmol/l)	0.12	0.246	1.13	0.92	1.37
Pre-pregnancy BMI (kg)	0.04	0.255	1.04	0.97	1.12
GWG (kg)	− 0.03	0.366	0.98	0.92	1.03
> 3 children (yes)	0.07	0.879	1.07	0.45	2.54
Low education (yes)	0.25	0.476	1.28	0.65	2.55
1.degree relatives w/diabetes (yes)	0.66	0.070	1.93	0.95	3.91
GDM before index pregn (yes)	1.18	0.031	3.24	1.11	9.45
Glucose-lowering drugs * GDM before index pregn	− 1.96	0.009	0.14	0.03	0.61

The model includes the covariates (odds ratio (OR) with 95% CI): age, ethnicity, glucose-lowering drugs, fasting plasma glucose (FPG) and 2-h oral glucose tolerance test (OGTT) values in pregnancy, pre-pregnancy BMI, gestational weight gain (GWG), parity, education, first-degree relatives with diabetes, gestational diabetes mellitus (GDM) before index pregnancy, and the interaction term: Glucose-lowering drugs * GDM before index pregnancy

South Asian ethnicity [OR 7.05 (95% CI (2.94–16.92))], GDM before the index pregnancy [OR 3.24 (1.11, 9.45)], using glucose lowering drugs in pregnancy [2.87 (1.31–6.30)], higher age [OR 1.11 (1.02, 1.21)], and higher in-pregnancy fasting OGTT glucose [1.96 (1.16, 3.29)] had a higher likelihood of actionable HbA_1c_ (Table 2). The association was not statistically significant for in-pregnancy 2-h OGTT glucose, pre-pregnancy BMI, and first-degree relatives with diabetes. GWG, education, and parity were not associated with actionable HbA_1c_.

A sensitivity analysis replacing pre-pregnancy BMI with WHR was performed to link at-visit adiposity status to at-visit HbA_1c_ levels. Adiposity was here significantly associated with actionable HbA_1c_ (Additional file 2: Table S3).

## Discussion

Identifying women with prediabetes or early type 2 diabetes in a high-risk group of relatively young women post-GDM is important for the planning of a potential next pregnancy and for implementing appropriate measures to prevent the development of type 2 diabetes and its complications. Our study showed that prediabetes was highly prevalent and more so among South Asian than Nordic women. Both the HbA_1c_ and the compound diagnostic criteria detected a pattern of excess risk in the South Asian compared to the Nordic women. However, HbA_1c_ seemed to capture this ethnic difference just as well as other tests and speculatively better. Adding the measurement of FPG to HbA_1c_, identified more women at risk and might be an option to consider in women planning further pregnancies. Important predictors for actionable HbA_1c_ were South Asian ethnicity, GDM before index pregnancy, use of glucose-lowering drugs in pregnancy, higher age, and higher in-pregnancy FPG. Whilst pre-pregnancy BMI did not feature as a significant predictor, when it was replaced by actual WHR, obesity appeared as a key modifiable risk factor.

### Prevalence of prediabetes and diabetes

In our population, applying the ADA-HbA_1c_ cut-offs of 39 mmol/mol (5.7%) identified 58.3% and 22.2% of South Asian and Nordic women with prediabetes or diabetes, of whom most had pre-diabetes. Hence, only two South Asian or five Nordic women would need to be tested to identify one woman with an actionable ADA-HbA_1c_ result. The comparable percentages by the WHO-HbA_1c_ cut-offs of 42 mmol/mol (6.0%) were 25.8 and 6.5%.

Similar to our findings in the Nordic population, a few studies [23–25] have reported a prevalence of 16-19% (applying the ADA-HbA_1c_ cut-points) for the diagnosis of prediabetes or diabetes in mostly white women 1–2.5 years post-GDM. Comparable data in the South Asian population is, so far, scarce.

As current guidelines differ regarding which test to use as post-GDM screening [7, 9, 26], we also assessed the prevalence of prediabetes and diabetes including the OGTT data. We then found that 87.8% and 65.7% of the South Asian and Nordic qualified for this diagnosis. These high prevalence rates, however, seems rather unrealistic in terms of future workload for prevention. One reason for this increase may be that some women have isolated peaks of postprandial glucose that do not translate into higher HbA_1c_ or into long-term diabetes complications [10, 11]. Although some studies defend the use of OGTT as a predictor of diabetes outcomes, they do not apply to our younger prediabetic population [27, 28]. Another possibility is that, despite our best efforts, perhaps some did not fast for as long as requested before their OGTT, once again reiterating the importance of using tests which have better analytical performance and are less prone to pre-analytical factors, which may vary by ethnicity. Even so, both the use of stand-alone HbA_1c_ and different compound diagnostic criteria confirmed a higher prevalence among the South Asian than the Nordic women (Fig. 1).

Two previous studies performed in India and Ireland with similar design to ours [6, 13] also reported high (albeit somewhat lower than the present study) prevalence rates of prediabetes or diabetes post-GDM (by the compound diagnostic ADA-criteria), 57.7% and 18.4%, respectively. In these studies, the IADPSG criteria were used for diagnosing GDM, in contrast to our WHO 99 (4.8%) or modified IADPSG (93%) criteria with higher cut-off values for the diagnosis. This might explain our higher prevalence rates, as a higher cut-off values are associated with higher conversion rates to diabetes [14, 29]. Nevertheless, the comparable proportion of Nordic women that developed prediabetes or diabetes in our study was unexpectedly high (65.7% vs. 18.4% in the Irish study)), since the Irish women had similar age and higher mean BMI (32.4 vs 29.3 kg/m^2^) and waist circumference (100.1 vs 96.1 cm). A Finnish study [30] reported the prevalence of prediabetes or diabetes to be > 50% 6 years post-GDM, which is in broad accordance with our results, acknowledging the differences in the GDM criteria applied.

A high prevalence of diabetes short time after pregnancy in South Asian women is consistent with current litterature, reporting a cumulative conversion rate to diabetes of 10.5% and 22.0% within ~4 and 5 years post-GDM on the basis of OGTT data [6, 14]. In Western women, however, studies have indicated a lower cumulative conversion rate of 2.3% and 5.8% within 5 and ~10 years [13, 30]. Notwithstanding this, the early post-GDM development of diabetes in both populations, yet meaningfully lower by the HbA_1c,_ underscores the importance of annually screening for prediabetes and diabetes post-GDM.

### Comparing different modes for diagnosing prediabetes and diabetes with diagnosis based on OGTT

Our study resonates strongly with previous work [13, 14, 31], acknowledging that adding a measurement of FPG to HbA_1c_ increases the diagnostic sensitivity for prediabetes and diabetes significantly. We also found that more women were identified by applying the ADA rather than the WHO-IEC criteria. Interestingly, the benefits of adding HbA_1c_ to FPG was only significant in the South Asian group. This finding emphasise that HbA_1c_ may efficiently capture the pattern of excess risk in South Asians, and is consistent with studies showing higher HbA_1c_ levels in South Asian than white women despite lower FPG [32]. Accordingly, this could partially explain the difference in HbA_1c_ between the ethnic groups for the best performance of the combined FPG and HbA_1c_ in our study (South Asian group 5.6 mmol/l and 38 mmol/mol (5.6%) vs 5.6 mmol/l and 37 mmol/mol (5.5%) in the Nordic group) (Additional file 2: Table S2), again with the assumption that OGTT is gold standard, which can now be strongly debated.

Whether adding FPG to HbA_1c_ identifies more women at risk for high glucose levels in subsequent pregnancies, and thereby adverse pregnancy outcomes [33–35], are not answered by our study and deserve further studies. In clinical practice, we support the use of HbA_1c_ testing for women post-GDM.

### Predictors for prediabetes or diabetes by ethnicity

In our multivariate analysis, South Asian ethnicity was the strongest risk factor for an actionable HbA_1c_ post-GDM. Although this finding was not unexpected and is supported by recent litterature, these studies all held the assumption that OGTT is gold standard for the diagnosis of prediabetes or diabetes [14, 36, 37]. To the best of our knowledge, this is the first study that sought to identify determinants for actionable ADA-HbA_1c_. And by doing so, it was reassuring to see that the predictors for glucose deterioration were consistent regardless of criteria used [30, 36, 38] (Table 2).

Of note, intake of glucose-lowering drugs did not impact the risk of actionable HbA_1c_ in women with GDM before index pregnancy, contrary to findings in women without GDM before index pregnancy, emphasizing the strong impact of previous GDM itself as a risk factor. This finding indicates that a GDM-pregnancy may result in a severe deterioration of the glucose metabolism, as stated by others [39], fitting with the high GDM recurrence rate in following pregnancies [40].

Overweight and obesity are well known risk factors for prediabetes and diabetes, and in our study 86% (82/95) of the South Asians, and 81% (17/21) of the Nordic women identified with actionable HbA_1c_ were either overweight or obese before the pregnancy. Type 2 diabetes is strongly associated with excess total and ectopic fat in all ethnicities, and the greater risk in South Asians may be linked to larger visceral fat and lower skeletal muscle mass for a given BMI [41]. Accordingly, we found larger WHR in South Asian than in Nordic women at similar BMI, and an increased risk for actionable HbA_1c_ by WHR, but not by BMI. There is also an emerging suggestion that South Asians have more rapid genetically-determined beta cell deterioration [42]; consistent with our findings that the increased risk of actionable HbA_1c_ among South Asians was not greatly attenuated after adjustment for WHR. Whatever the mechanism, we strongly encourage weight redcution in overweight women post-GDM, as modest weight loss can prevent diabetes [43] and greater weight loss can reverse diabetes [44]. Such weight loss reduces liver fat, and may improve beta cell function in whites, at least [45], though weight loss led to similar or even greater diabetes remission in a Qatari population [46].

### Strengths and limitations

The major strengths of this multicentre study is the inclusion of a relatively large sample size from two different ethnic groups living in the same area and cared for in the same healthcare setting. Futhermore, the application of different definitions for prediabetes that might make comparison between studies easier is also a major strength. Finally, reporting on ethnic difference in HbA_1c_-based prevalence of prediabetes and diabetes post-GDM is novel and important, especially as rates of diabetes and obesity are rasing worldwide, especially in “metabolically higher risk” South Asians communities.

Our study has limitations. First, we only recruited women referred to hospital for treatment of GDM. Our findings are, therefore, not applicable to women with diet-treated GDM cared for in primary healthcare.

Second, we cannot exclude a selection bias due to the low participation rate (45% and 14% in the South Asian and the Nordic groups). We, therefore, compared significant baseline characteristics in the regression analysis of women who did vs. a randomly selected subgroup of women who did not participate in the study (100 South Asian and 100 Nordic women) (Additional file 2: Table S4). Among the South Asian women no difference in age, pre-pregnancy BMI, in-pregnancy glucose values, the use of glucose-lowering drugs, GDM before index pregnancy, or first-degree relatives with diabetes were found. The participating Nordic women were older than non-participants, but the other characteristics did not differ. The older age among participating Nordic women might have led to an overestimation of the proportion of women with actionable HbA_1c_ in this group. Third, differences in the recruitment procedures may have introduced a selection bias between the ethnic groups. After sending invitation letters to eligible women, only South Asian women received a telephone invitation to enhance recruitment. It is therefore possible that Nordic women with higher in-pregnancy glucose levels may have been more likely to respond to the invitation. Speaking against this is the fact that a higher percentage of South Asian than Nordic women were using glucose-lowering drugs in pregnancy and by minimal differences in the in-pregnancy glucose levels between the ethnic groups (Table 1). Fourth, our analyses did not include assessments of dietary habits and physical activity that may have important impact on the prevalence rates in the two groups. Our study had limited power to detect ethnic difference in the prevalence of diabetes. Finally, causality cannot be inferred from a cross-sectional study, and a single measurement of HbA_1c_ or OGTT is not sufficient for a diagnosis of diabetes in asymptomatic women.

## Conclusions

We found high prevalence rates of actionable HbA_1c_ in women with GDM 1–3 years previously. Both the HbA_1c_ and the established compound diagnostic criteria for prediabetes including OGTT measurements detected a pattern of excess risk in the South Asian compared to the Nordic women. The HbA_1c_-based criteria captured the ethnic difference best. Significant risk factors for actionable HbA_1c_ were South Asian ethnicity, GDM before index pregnancy, use of glucose-lowering drugs in pregnancy, higher age, and in-pregnancy fasting glucose levels; all factors linked downstream to excess adiposity in both ethnicities. In terms of public health recommendations, our results confirm the need for regular screening of prediabetes and diabetes post-GDM, and support the use of HbA_1c_ to improve women’s adherence to follow-ups and better predict long-term risks after GDM, particularly in South Asian communities.

## Supplementary Information

**Additional file 1: Fig. S1.** Participant flow-chart. **Fig. S2.** HbA_1c_ and OGTT-based prevalence (95% CI) of prediabetes and diabetes by ethnicity and different diagnostic criteria.

**Additional file 2: Table S1.** Performance of FPG, HbA1c, and FPG & HbA1c by ethnicity for diagnosing prediabetes and diabetes compared to different diagnostic criteria. **Table S2.** Performance of different combinations of FPG and HbA_1c_ cut-offs for diagnosing prediabetes or diabetes compared to ADA criteria. **Table S3.** Logistic regression analysis showing odds ratio (OR) with 95 % CI for actionable ADA-HbA1c (defined as prediabetes or diabetes) after adjusting for covariates. **Table S4.** The participating and non-participating women’s characteristics by ethnicity.

## Data Availability

All data generated or analysed during this study are included in this published article and its supplementary information files.

## Abbreviations

FPG: Fasting plasma glucose
GDM: Gestational diabetes mellitus
GWS: Gestational weight gain
IADPSG: International Association of Diabetes and Pregnancy Study Group
OGTT: Oral glucose tolerance test
WHO-IEC: World Health Organization-International Expert Committee

## References

[1] Vounzoulaki E, Khunti K, Abner SC, Tan BK, Davies MJ, Gillies CL (2020). Progression to type 2 diabetes in women with a known history of gestational diabetes: systematic review and meta-analysis. BMJ.

[2] Dennison RA, Chen ES, Green ME, Legard C, Kotecha D, Farmer G, Sharp SJ, Ward RJ, Usher-Smith JA, Griffin SJ (2021). The absolute and relative risk of type 2 diabetes after gestational diabetes: a systematic review and meta-analysis of 129 studies. Diabetes Res Clin Pract.

[3] Das Gupta R, Gupta S, Das A, Biswas T, Haider MR, Sarker M (2018). Ethnic predisposition of diabetes mellitus in the patients with previous history of gestational diabetes mellitus: a review. Expert Rev Endocrinol Metab.

[4] Zhu Y, Zhang C (2016). Prevalence of gestational diabetes and risk of progression to type 2 diabetes: a global perspective. Curr Diab Rep.

[5] Jenum AK, Mørkrid K, Sletner L, Vangen S, Torper JL, Nakstad B, Voldner N, Rognerud-Jensen OH, Berntsen S, Mosdøl A (2012). Impact of ethnicity on gestational diabetes identified with the WHO and the modified International Association of Diabetes and Pregnancy Study Groups criteria: a population-based cohort study. Eur J Endocrinol.

[6] Goyal A, Gupta Y, Kalaivani M, Sankar MJ, Kachhawa G, Bhatla N, Gupta N, Tandon N (2018). Long term (>1 year) postpartum glucose tolerance status among Indian women with history of gestational diabetes mellitus (GDM) diagnosed by IADPSG criteria. Diabetes Res Clin Pract.

[7] Norwegian Directorate of Health.no. Nasjonal faglig retningslinje: Svangerskapsdiabetes [National guidelines gestational diabetes mellitus]. 2018. Available from: https://www.helsedirektoratet.no/retningslinjer/svangerskapsdiabetes. Accessed 30 May 2022.

[8] American Diabetes Association Professional Practice C (2022). 15. Management of diabetes in pregnancy: standards of medical care in diabetes—2022. Diabetes Care.

[9] National Insititute for Health and Care Excellence (NICE) (2012). Type 2 diabetes: prevention in people at high risk.

[10] Colagiuri S, Lee CMY, Wong TY, Balkau B, Shaw JE, Borch-Johnsen K, Group D-CW (2011). Glycemic thresholds for diabetes-specific retinopathy: implications for diagnostic criteria for diabetes. Diabetes Care.

[11] Vistisen D, Witte DR, Brunner EJ, Kivimäki M, Tabák A, Jørgensen ME, Færch K (2018). Risk of cardiovascular disease and death in individuals with prediabetes defined by different criteria: the Whitehall II study. Diabetes Care.

[12] Di Angelantonio E, Gao P, Khan H, Butterworth AS, Wormser D, Kaptoge S, Kondapally Seshasai SR, Thompson A, Sarwar N, Willeit P (2014). Glycated hemoglobin measurement and prediction of cardiovascular disease. JAMA.

[13] Noctor E, Crowe C, Carmody LA, Avalos GM, Kirwan B, Infanti JJ, O'Dea A, Gillespie P, Newell J, McGuire B (2013). ATLANTIC DIP: simplifying the follow-up of women with previous gestational diabetes. Eur J Endocrinol.

[14] Gupta Y, Kapoor D, Desai A, Praveen D, Joshi R, Rozati R, Bhatla N, Prabhakaran D, Reddy P, Patel A (2017). Conversion of gestational diabetes mellitus to future type 2 diabetes mellitus and the predictive value of HbA1c in an Indian cohort. Diabet Med.

[15] Zhang M, Zhou Y, Zhong J, Wang K, Ding Y, Li L (2019). Current guidelines on the management of gestational diabetes mellitus: a content analysis and appraisal. BMC Pregnancy Childbirth.

[16] World Health Organization.int (1999). Definition, diagnosis and classification of diabetes mellitus and its complications: report of a WHO consultation. Part 1, Diagnosis and classification of diabetes mellitus.

[17] The International Expert Committee (2009). International Expert Committee Report on the role of the A1C assay in the diagnosis of diabetes. Diabetes Care.

[18] American Diabetes Association Professional Practice C (2022). 2. Classification and diagnosis of diabetes: standards of medical care in diabetes—2022. Diabetes Care.

[19] World Health Organization. STEP surveillance. Section 4: Guide to physical measurements (step 2). Available from: https://www.who.int/ncds/surveillance/steps/Section%204%20Step%202%20Physical%20Measurements.pdf. Accessed 30 May 2022.

[20] World Health Organization (2011). Use of glycated haemoglobin (HbA1c) in diagnosis of diabetes mellitus: abbreviated report of a WHO consultation.

[21] Oldfield MD, Donley P, Walwyn L, Scudamore I, Gregory R (2007). Long term prognosis of women with gestational diabetes in a multiethnic population. Postgrad Med J.

[22] WHO expert consultation (2004). Appropriate body-mass index for Asian populations and its implications for policy and intervention strategies. Lancet.

[23] Picón MJ, Murri M, Muñoz A, Fernández-García JC, Gomez-Huelgas R, Tinahones FJ (2012). Hemoglobin A1c versus oral glucose tolerance test in postpartum diabetes screening. Diabetes Care.

[24] Claesson R, Ekelund M, Ignell C, Berntorp K (2014). Role of HbA1c in post-partum screening of women with gestational diabetes mellitus. J Clin Transl Endocrinol.

[25] Quansah DY, Gross J, Mbundu-Ilunga R, Puder JJ (2021). The utility of diagnostic tests in the detection and prediction of glucose intolerance in the early and late postpartum period in women after gestational diabetes: a longitudinal cohort study. Diabetol Metab Syndr.

[26] American Diabetes Association (2021). 14. Management of diabetes in pregnancy: standards of medical care in diabetes—2021. Diabetes Care.

[27] Group DS, on behalf of the European Diabetes Epidemiology G (2001). Glucose tolerance and cardiovascular mortality: comparison of fasting and 2-hour diagnostic criteria. Arch Intern Med.

[28] Meigs JB, Nathan DM, D’Agostino RB, Wilson PW (2002). Fasting and postchallenge glycemia and cardiovascular disease risk: the Framingham Offspring Study. Diabetes Care.

[29] Li Z, Cheng Y, Wang D, Chen H, Chen H, Ming W-K, Wang Z (2020). Incidence rate of type 2 diabetes mellitus after gestational diabetes mellitus: a systematic review and meta-analysis of 170,139 women. J Diabetes Res.

[30] Huopio H, Hakkarainen H, Pääkkönen M, Kuulasmaa T, Voutilainen R, Heinonen S, Cederberg H (2014). Long-term changes in glucose metabolism after gestational diabetes: a double cohort study. BMC Pregnancy Childbirth.

[31] Su X, Zhang Z, Qu X, Tian Y, Zhang G (2014). Hemoglobin A1c for diagnosis of postpartum abnormal glucose tolerance among women with gestational diabetes mellitus: diagnostic meta-analysis. PLoS One.

[32] Herman WH, Ma Y, Uwaifo G, Haffner S, Kahn SE, Horton ES, Lachin JM, Montez MG, Brenneman T, Barrett-Connor E (2007). Differences in A1C by race and ethnicity among patients with impaired glucose tolerance in the Diabetes Prevention Program. Diabetes Care.

[33] HAPO study Cooperative Research Group (2008). Hyperglycemia and adverse pregnancy outcomes. N Engl J Med.

[34] Simmons D (2021). Paradigm shifts in the management of diabetes in pregnancy: the importance of type 2 diabetes and early hyperglycemia in pregnancy. Diabetes Care.

[35] Brand JS, West J, Tuffnell D, Bird PK, Wright J, Tilling K, Lawlor DA (2018). Gestational diabetes and ultrasound-assessed fetal growth in South Asian and White European women: findings from a prospective pregnancy cohort. BMC Med.

[36] Benhalima K, Van Crombrugge P, Moyson C, Verhaeghe J, Vandeginste S, Verlaenen H, Vercammen C, Maes T, Dufraimont E, De Block C (2019). Prediction of glucose intolerance in early postpartum in women with gestational diabetes mellitus based on the 2013 WHO criteria. J Clin Med.

[37] Rayanagoudar G, Hashi AA, Zamora J, Khan KS, Hitman GA, Thangaratinam S (2016). Quantification of the type 2 diabetes risk in women with gestational diabetes: a systematic review and meta-analysis of 95,750 women. Diabetologia.

[38] Ratner RE (2007). Prevention of type 2 diabetes in women with previous gestational diabetes. Diabetes Care.

[39] Barbour LA (2019). Metabolic culprits in obese pregnancies and gestational diabetes mellitus: big babies, big twists, big picture. Diabetes Care.

[40] Schwartz N, Nachum Z, Green MS (2015). The prevalence of gestational diabetes mellitus recurrence—effect of ethnicity and parity: a metaanalysis. Am J Obstet Gynecol.

[41] Sattar N, Gill JMR (2015). Type 2 diabetes in migrant south Asians: mechanisms, mitigation, and management. Lancet Diabetes Endocrinol.

[42] Siddiqui MK, Anjana RM, Dawed AY, Martoeau C, Srinivasan S, Saravanan J, Madanagopal SK, Veluchamy A, Pradeepa R, Sattar N (2022). Young onset diabetes in Asian Indians is associated with lower measured and genetically determined beta-cell function: An INSPIRED study. Diabetologia.

[43] Knowler WC, Barrett-Connor E, Fowler SE, Hamman RF, Lachin JM, Walker EA, Nathan DM, Diabetes Prevention Program Research G (2002). Reduction in the incidence of type 2 diabetes with lifestyle intervention or metformin. N Engl J Med.

[44] Lean MEJ, Leslie WS, Barnes AC, Brosnahan N, Thom G, McCombie L, Peters C, Zhyzhneuskaya S, Al-Mrabeh A, Hollingsworth KG (2018). Primary care-led weight management for remission of type 2 diabetes (DiRECT): an open-label, cluster-randomised trial. Lancet.

[45] Taylor R, Al-Mrabeh A, Sattar N (2019). Understanding the mechanisms of reversal of type 2 diabetes. Lancet Diabetes Endocrinol.

[46] Taheri S, Zaghloul H, Chagoury O, Elhadad S, Ahmed SH, El Khatib N, Amona RA, El Nahas K, Suleiman N, Alnaama A (2020). Effect of intensive lifestyle intervention on bodyweight and glycaemia in early type 2 diabetes (DIADEM-I): an open-label, parallel-group, randomised controlled trial. Lancet Diabetes Endocrinol.

