# Supplementary material for: High prevalence and significant ethnic differences in actionable HbA1C after gestational diabetes mellitus in women living in Norway
Source: BMC Med. 2022 Sep 23;20:318. doi: 10.1186/s12916-022-02515-w (PMC9502889; doi:10.1186/s12916-022-02515-w)
Supplement: Supplementary file 1 — Additional file 1: Fig. S1. Participant flow-chart. Fig. S2. HbA1c and OGTT-based prevalence (95% CI) of prediabetes and diabetes by ethnicity and different diagnostic criteria. [file 12916_2022_2515_MOESM1_ESM.docx]

**Additional file 1:**

Fig. S1. Participant flow-chart


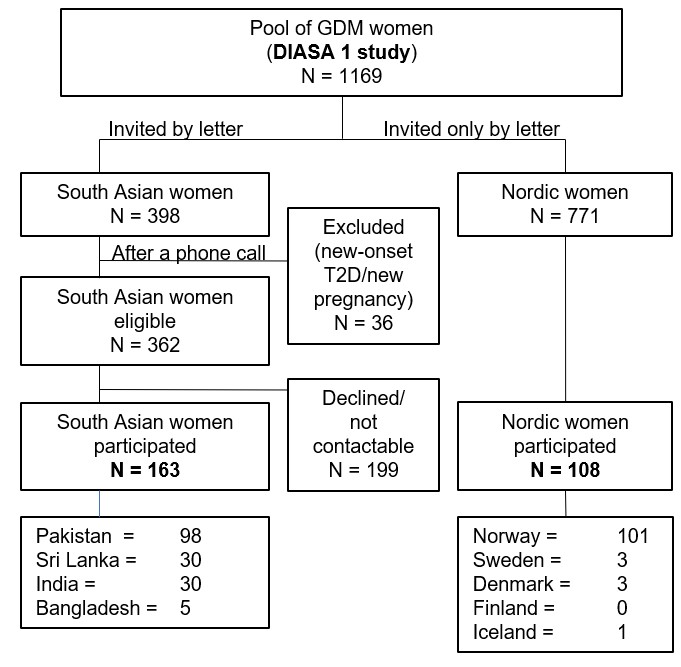


Fig. S2. HbA_1c_ and OGTT-based prevalence (95% CI) of prediabetes and diabetes by ethnicity and different diagnostic criteria


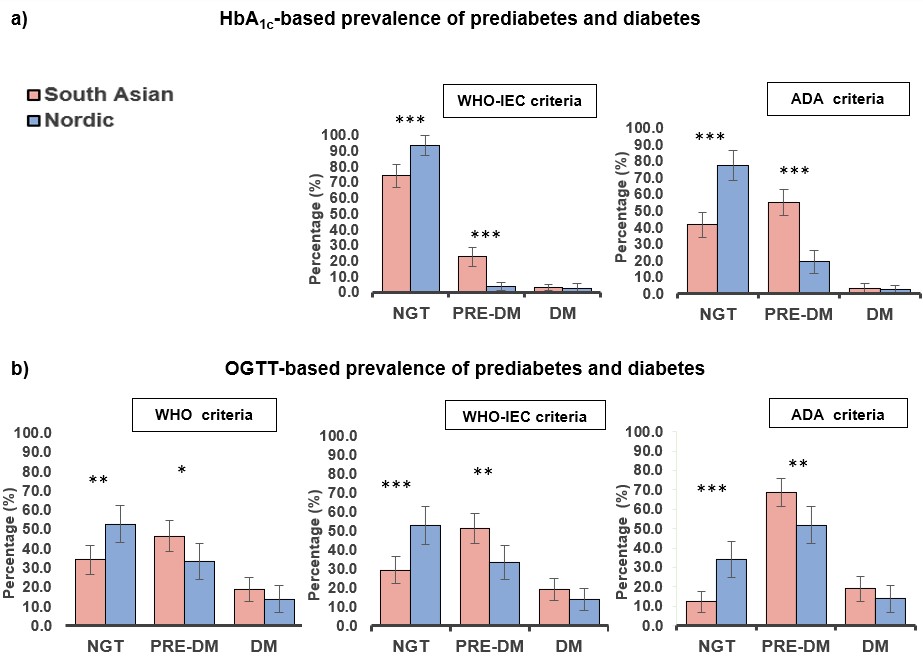


1. HbA_1c_-based prevalence with 95% CI of normal glucose tolerance (NGT), prediabetes (PRE-DM) and diabetes (DM) by ethnicity, using the WHO-IEC (prediabetes: HbA_1c_ 42-47 mmol/mol (6.0-6.4%)) and the ADA criteria (prediabetes: HbA_1c_ 39-47 mmol/mol (5.7-

6.4%)).

1. OGTT-based prevalence with 95% CI of NGT, PRE-DM and DM by ethnicity, using the

WHO (prediabetes: fasting plasma glucose (FPG) 6.1-6.9 mmol/L and/or 2-h plasma glucose

7.8-11.0 mmol/L), WHO-IEC (prediabetes: FPG 6.1-6.9 mmol/L and/or 2-h plasma glucose

7.8-11.0 mmol/L and/or HbA_1c_ 42-47 mmol/mol (6.0-6.4%)), and ADA criteria (prediabetes: FPG 5.6-6.9 mmol/L and/or 2-h plasma glucose 7.8-11.0 mmol/L and/or HbA_1c_ 39-47 mmol/mol (5.7-6.4%)).

* p-value ≤ 0.05, ** p-value ≤ 0.01, *** p-value ≤ 0.001
