# Supplementary material for: High prevalence and significant ethnic differences in actionable HbA1C after gestational diabetes mellitus in women living in Norway
Source: BMC Med. 2022 Sep 23;20:318. doi: 10.1186/s12916-022-02515-w (PMC9502889; doi:10.1186/s12916-022-02515-w)
Supplement: Supplementary file 2 — Additional file 2: Table S1. Performance of FPG, HbA1c, and FPG & HbA1c by ethnicity for diagnosing prediabetes and diabetes compared to different diagnostic criteria. Table S2. Performance of different combinations of FPG and HbA1c cut-offs for diagnosing prediabetes or diabetes compared to ADA criteria. Table S3. Logistic regression analysis showing odds ratio (OR) with 95 % CI for actionable ADA-HbA1c (defined as prediabetes or diabetes) after adjusting for covariates. Table S4. The participating and non-participating women’s characteristics by ethnicity. [file 12916_2022_2515_MOESM2_ESM.docx]

**Additional file 2:**

Table S1. Performance of FPG, HbA_1c,_ and FPG & HbA_1c_ by ethnicity for diagnosing prediabetes and diabetes compared to different diagnostic criteria

| **Characteristics** | **South Asian women % (95% CI)** | | |  | **Nordic women % (95% CI)** | | |
| --- | --- | --- | --- | --- | --- | --- | --- |
|  |  |  |  |  | |  |  |
|  | **WHO criteria** | **WHO-IEC**  **criteria** | **ADA**  **Criteria** | **WHO criteria** | | **WHO-IEC**  **criteria** | **ADA**  **Criteria** |
| **PREDIABETES OR DIABETES** | | | | | | | |
| **FPG** |  | | | | | | |
| Sensitivity | 41 (32-51) | 38 (29-48) | 71 (63-79) | 47 (33-62) | | 47 (33-62) | 75 (63-84) |
| Specificity | 100 (94-100) | 100 (93-100) | 100 (83-100) | 100 (94-100) | | 100 (93-100) | 100 (83-100) |
| PPV | 100 | 100 | 100 | 100 | | 100 | 100 |
| NPV | 47 (43-51) | 40 (37-44) | 33 (27-39) | 68 (62-73) | | 68 (62-73) | 67 (58-75) |
| **HbA_1c_** |  | | | | | | |
| Sensitivity | 32 (23-41) | 37 (28-46) | 66 (58-74) | 14 (6-26) | | 14 (6-26) | 34 (23-46) |
| Specificity | 86 (74-94) | 100 (93-100) | 100 (83-100) | 100 (94-100) | | 100 (94-100) | 100 (91-100) |
| PPV | 81 (68-90) | 100 | 100 | 100 | | 100 | 100 |
| NPV | 40 (36-44) | 40 (36-43) | 29 (25-34) | 56 (54-59) | | 56 (54-59) | 44 (40-48) |
| **FPG & HbA_1c_** |  | | | | | | |
| Sensitivity |  | 55 (45-64) | 86 (79-91) |  | | 49 (35-63) | 83 (72-91) |
| Specificity |  | 100 (93-100) | 100 (83-100) |  | | 100 (94-100) | 100 (91-100) |
| PPV |  | 100 | 100 |  | | 100 | 100 |
| NPV |  | 48 (43-53) | 50 (40-60) |  | | 69 (63-74) | 76 (65-84) |
| **DIABETES** | | | | | | | |
| **FPG** |  | | | | | | |
| Sensitivity | 42 (25-61) | | | 47 (21-73) | | | |
| Specificity | 100 (97-100) | | | 100 (96-100) | | | |
| PPV | 100 | | | 100 | | | |
| NPV | 88 (84-91) | | | 92 (88-95) | | | |
| **HbA_1c_** |  | | | | | | |
| Sensitivity | 16 (5-34) | | | 20 (4-48) | | | |
| Specificity | 100 (97-100) | | | 100 (96-100) | | | |
| PPV | 100 | | | 100 | | | |
| NPV | 84 (81-86) | | | 89 (86-91) | | | |
| **FPG & HbA_1c_** |  | | | | | | |
| Sensitivity | 48 (30-67) | | | 53 (27-79) | | | |
| Specificity | 100 (97-100) | | | 100 (96-100) | | | |
| PPV | 100 | | | 100 | | | |
| NPV | 90 (85-94) | | | 94 (87-97) | | | |

FPG, fasting plasma glucose; PPV, positive predictive value; NPV, negative predictive value

Table S2. Performance of different combinations of FPG and HbA_1c_ cut-offs for diagnosing prediabetes or diabetes compared to ADA criteria

| **Ethnicity** | **FBG**  (mmol/l) | **HbA_1c_ (mmol/mol)** | **No of women meeting criteria** n (%) | **Sensitivity**  % (95% CI) | **Specificity**  % (95% CI) | **PPV**  % (95% CI) | **NPV**  % (95% CI) |
| --- | --- | --- | --- | --- | --- | --- | --- |
| South Asian | **5.5** | **36** | 135 (83) | 94 (89-98) | 45 (23-68) | 92 (89-95) | 53 (33-72) |
| Nordic |  |  | 65 (60) | 92 (83-97) | 49 (32-66) | 77 (71-83) | 75 (57-87) |
| South Asian | **5.6** | **36** | 134 (82) | 94 (88-97) | 50 (27-73) | 93 (90-95) | 53 (34-71) |
| Nordic |  |  | 65 (60) | 92 (83-97) | 62 (45-78) | 82 (75-88) | 79 (63-90) |
| South Asian | **5.7** | **36** | 131 (80) | 92 (86-96) | 50 (27-73) | 93 (89-95) | 46 (29-63) |
| Nordic |  |  | 62 (57) | 87 (77-94) | 62 (45-78) | 82 (74-87) | 72 (57-83) |
| South Asian | **5.5** | **37** | 130 (80) | 91 (85-95) | 60 (36-81) | 94 (90-96) | 48 (33-63) |
| Nordic |  |  | 64 (59) | 90 (81-96) | 68 (50-82) | 84 (77-90) | 78 (63-88) |
| South Asian | **5.6** | **37** | 129 (79) | 90 (84-95) | 75 (51-91) | 96 (92-98) | 52 (38-65) |
| Nordic |  |  | 64 (59) | **90 (81-96)** | **84 (68-94)** | **91 (84-96)** | **82 (68-90)** |
| South Asian | **5.7** | **37** | 125 (77) | 87 (81-92) | 75 (51-91) | 96 (92-98) | 45 (34-58) |
| Nordic |  |  | 61 (56) | 86 (76-93) | 84 (68-94) | 91 (83-96) | 76 (63-85) |
| South Asian | **5.5** | **38** | 128 (79) | 90 (83-94) | 80 (56-94) | 97 (93-99) | 52 (39-64) |
| Nordic |  |  | 61 (56) | 86 (76-93) | 73 (56-86) | 86 (78-91) | 73 (60-83) |
| South Asian | **5.6** | **38** | 127 (78) | **89 (82-93)** | **95 (75-100)** | **99 (95-100)** | **54 (43-66)** |
| Nordic |  |  | 60 (56) | 85 (74-92) | 92 (78-98) | 95 (87-98) | 76 (64-84) |
| South Asian | **5.7** | **38** | 121 (74) | 85 (78-90) | 95 (75-100) | 99 (95-100) | 46 (37-56) |
| Nordic |  |  | 57 (53) | 83 (73-90) | 92 (78-98) | 96 (88-99) | 71 (60-80) |
| South Asian | **5.5** | **39** | 124 (76) | 87 (80-92) | 85 (62-97) | 98 (94-99) | 47 (36-59) |
| Nordic |  |  | 60 (56) | 85 (74-92) | 81 (65-92) | 90 (81-94) | 73 (61-83) |
| South Asian | **5.6** | **39** | 123 (75) | 86 (79-91) | 100 (82-100) | 100 | 50 (40-60) |
| Nordic |  |  | 59 (55) | 83 (72-91) | 100 (91-100) | 100 | 76 (65-84) |
| Nordic | **5.7** | **39** | 115 (71) | 80 (73-87) | 100 (83-100) | 100 | 42 (34-50) |
| South Asian |  |  | 55 (51) | 77 (66-87) | 100 (91-100) | 100 | 70 (60-78) |
| South Asian | **5.5*** | **42*** | 88 (54) | 77 (68-84) | 46 (32-61) | 77 (72-82) | 45 (34-56) |
| Nordic |  |  | 38 (35) | 75 (60-86) | 58 (44-71) | 61 (53-69) | 72 (60-81) |
| South Asian | **5.6*** | **42*** | 85 (52) | 74 (65-82) | 52 (37-67) | 79 (73-84) | 46 (36-56) |
| Nordic |  |  | 36 (33) | 71 (56-83) | 70 (57-82) | 68 (58-77) | 72 (63-81) |
| South Asian | **5.7*** | **42*** | 79 (48) | 41 (31-52) | 63 (52-73) | 51 (42-60) | 53 (47-59) |
| Nordic |  |  | 33 (31) | 65 (50-78) | 72 (58-83) | 67 (57-77) | 69 (60-77) |
| South Asian | **6.1*** | **42*** | 63 (39) | 50 (40-60) | 54 (44-64) | 52 (45-59) | 53 (46-59) |
| Nordic |  |  | 25 (23) | 49 (35-63) | 100 (94-100) | 100 | 69 (62-74) |

* Compared to WHO-IEC criteria. FPG, fasting plasma glucose

Table S3. Logistic regression analysis showing odds ratio (OR) with 95 % CI for actionable ADA-HbA_1c_ (defined as prediabetes or diabetes) after adjusting for covariates

| **Risk factors** | **Estimated coefficients** | **p-value** | **OR** | **OR (95% C.I.)**  **Lower Upper** | |
| --- | --- | --- | --- | --- | --- |
| Age (years) | 0.09 | 0.040 | 1.09 | 1.00 | 1.19 |
| Ethncity | 1.76 | <0.001 | 5.78 | 2.47 | 13.53 |
| Glucose-lowering drugs (yes) | 1.17 | <0.001 | 3.23 | 1.49 | 7.00 |
| FPG at OGTT in pregnancy (mmol/l) | 0.62 | 0.015 | 1.87 | 1.13 | 3.08 |
| 2-h OGTT glucose in pregnancy (mmol/l) | 0.11 | 0.246 | 1.12 | 0.93 | 1.36 |
| Waist-to-hip ratio (per 0.10 cm) | 0.47 | 0.037 | 1.61 | 1.03 | 2.51 |
| GWG (kg) | - 0.03 | 0.231 | 0.97 | 0.92 | 1.02 |
| > 3 children (yes) | 0.11 | 0.801 | 1.12 | 0.48 | 2.60 |
| Low education (yes) | 0.16 | 0.657 | 1.17 | 0.59 | 2.31 |
| 1.degree relatives w/diabetes (yes) | 0.62 | 0.090 | 1.85 | 0.91 | 3.76 |
| GDM before index pregn (yes) | 1.11 | 0.042 | 3.05 | 1.04 | 8.91 |
| Glucose-lowering drugs *  GDM before index pregn | -1.96 | 0.008 | 0.14 | 0.03 | 0.60 |

The model includes the covariates: age, ethnicity, glucose-lowering drugs, fasting plasma glucose (FPG) and 2-h oral glucose tolerance test (OGTT) values in pregnancy, waist-to-hip ratio, gestational weight gain (GWG), parity, education, first-degree relatives with diabetes, gestational diabetes mellitus (GDM) before index pregnancy, and the interaction term: Glucose-lowering drugs * GDM before index pregnancy

Table S4. The participating and non-participating women’s characteristics by ethnicity

|  | **South Asian** | |  | **Nordic** | |  |
| --- | --- | --- | --- | --- | --- | --- |
|  | Participants  n = 163 | Non-participants  n = 100 | p-value | Participants  n = 108 | Non-participants  n = 100 | p-value |
| Pre-pregnancy age (years) | 33.4 (4.1) | 33.0 (4.2) | 0.447 | 35.2 (4.7) | 33.3 (6.2) | 0.013 |
| Self-reported pre-pregnancy weight (kg) | 70.6 (13.8) | 69.7 (15.9) | 0.629 | 79.3 (18.0) | 81.5 (18.7) | 0.388 |
| Pre-pregnancy BMI (based on self-reported weight) | 27.6 (5.0) | 27.1 (5.7) | 0.456 | 28.4 (6.2) | 29.4 (5.9) | 0.236 |
| Pregnancy FPG at OGTT | 5.5 (0.7) | 5.4 (0.7) | 0.262 | 5.3 (0.7) | 5.5 (1.0) | 0.094 |
| Pregnancy 2-h OGTT glucose | 9.3 (1.8) | 9.4 (1.9) | 0.669 | 9.0 (1.7) | 9.2 (1.3) | 0.345 |
| Insulin ± Metformin use in pregnancy | 89 [55] | 55 [55] | 0.950 | 40 [37] | 41/99 [41] | 0.556 |
| Insulin use in pregnancy | 65 [40] | 40 [40] | 0.987 | 34 [31] | 36/99 [36] | 0.447 |
| GDM prior to the index pregnancy | 51 [32] | 27 [27] | 0.392 | 24 [22] | 14 [14] | 0.136 |
| 1.degree relatives w/diabetes | 115/155 [74] | 69/92 [75] | 0.862 | 22/91 [24] | 20/80 [25] | 0.531 |

Data presented as mean (SD) or number (n) [%] for women who participated vs. a subgroup of women who did not participate in the study.

FPG, fasting plasma glucose. GDM, gestational diabetes mellitus. OGTT, oral glucose tolerance test
